# Bis(1,10-phenanthroline-κ^2^ *N*,*N*′)(sulfato-κ*O*)copper(II) ethanol monosolvate

**DOI:** 10.1107/S1600536813026093

**Published:** 2013-09-28

**Authors:** Natthaya Meundaeng, Timothy J. Prior, Apinpus Rujiwatra

**Affiliations:** a Department of Chemistry, Faculty of Science, Chiang Mai University, Chiang Mai 50200, Thailand; b Department of Chemistry, University of Hull, Cottingham Road, Hull, HU6 7RX, England

## Abstract

The crystal structure of the title compound, [Cu(SO_4_)(C_12_H_8_N_2_)_2_]·C_2_H_5_OH, arises from the assembly of the neutral complex [Cu(SO_4_)(C_12_H_8_N_2_)_2_] and an ethanol solvent mol­ecule. The Cu^II^ ion is five-coordinate, surrounded by two pairs of N atoms from two independent *N*,*N*′-chelating 1,10-phenanthroline ligands, and one O atom of monodentate sulfate ligand, in a distorted trigonal-bipyramidal fashion. Spatial orientation of the ligands and the assembly in the solid state are stabilized by the C—H⋯O hydrogen-bonding inter­actions, established between the O atoms (from the sulfate ligand and the ethanol mol­ecule) and the neighbouring 1,10-phenanthroline mol­ecules. There is also an offset face-to-face π–π stacking between the 1,10-phenanthroline ligands. The ethanol solvent mol­ecule is disordered over two orientations in the ratio 0.663 (10):0.337 (10). The crystal examined was subject to racemic twinning and the refined twin fraction was 0.346 (19).

## Related literature

Zhong has published many similar compounds with different solvent systems, see, for example: Zhong (2011*a* ▶,*b* ▶, 2012 ▶); Zhong & Cao (2013 ▶). For a similar centrosymmetric compound featuring 2,2′-bi­pyridine and bidentate sulfate, see: Wojciechowska *et al.* (2011 ▶). For similar compounds of different first-row transition metals, see, for example: Zhu *et al.* (2006 ▶); Zhong *et al.* (2009 ▶).
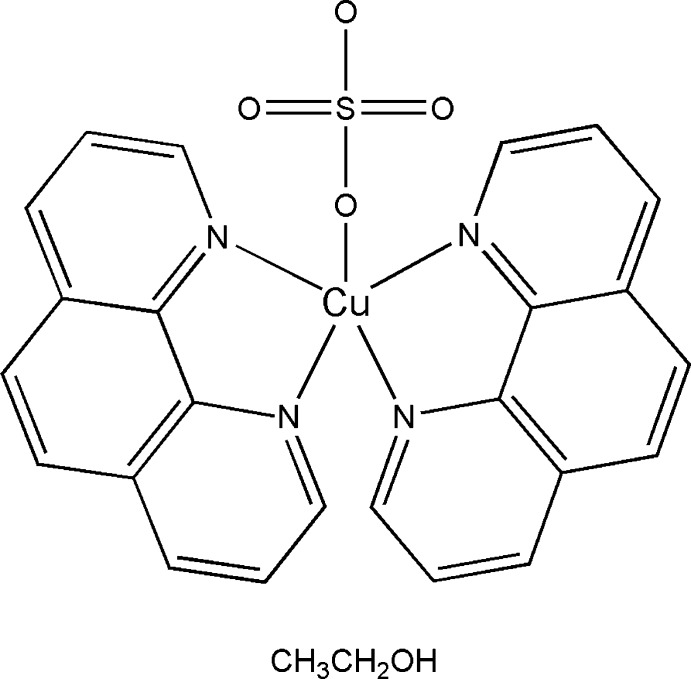


## Experimental

### 

#### Crystal data

[Cu(SO_4_)(C_12_H_8_N_2_)_2_]·C_2_H_6_O
*M* *_r_* = 564.06
Monoclinic, 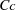

*a* = 17.5488 (14) Å
*b* = 11.9360 (11) Å
*c* = 13.0663 (9) Å
β = 120.664 (5)°
*V* = 2354.2 (3) Å^3^
*Z* = 4
Mo *K*α radiation
μ = 1.06 mm^−1^
*T* = 150 K
0.32 × 0.24 × 0.12 mm

#### Data collection

Stoe IPDS2 diffractometer
Absorption correction: numerical (*X-AREA*; Stoe & Cie, 2002 ▶) *T*_min_ = 0.727, *T*_max_ = 0.883
10087 measured reflections
5771 independent reflections
4259 reflections with *I* > 2σ(*I*)
*R*_int_ = 0.070

#### Refinement

*R*[*F* ^2^ > 2σ(*F* ^2^)] = 0.058
*wR*(*F* ^2^) = 0.160
*S* = 1.01
5771 reflections
331 parameters
8 restraints
H-atom parameters constrained
Δρ_max_ = 1.02 e Å^−3^
Δρ_min_ = −1.09 e Å^−3^
Absolute structure: Flack (1983 ▶), 2594 Friedel pairs
Absolute structure parameter: 0.346 (19)

### 

Data collection: *X-AREA* (Stoe & Cie, 2002 ▶); cell refinement: *X-AREA*; data reduction: *X-AREA*; program(s) used to solve structure: *SHELXS86* (Sheldrick, 2008 ▶); program(s) used to refine structure: *SHELXL97* (Sheldrick, 2008 ▶); molecular graphics: *DIAMOND* (Brandenburg, 1999 ▶); software used to prepare material for publication: *publCIF* (Westrip, 2010 ▶) and *PLATON* (Spek, 2009 ▶).

## Supplementary Material

Crystal structure: contains datablock(s) I, New_Global_Publ_Block. DOI: 10.1107/S1600536813026093/gg2128sup1.cif

Structure factors: contains datablock(s) I. DOI: 10.1107/S1600536813026093/gg2128Isup2.hkl

Additional supplementary materials:  crystallographic information; 3D view; checkCIF report

## Figures and Tables

**Table 1:** Hydrogen-bond geometry (Å, °)

*D*—H⋯*A*	*D*—H	H⋯*A*	*D*⋯*A*	*D*—H⋯*A*
C3—H3⋯O2^i^	0.95	2.48	3.389 (9)	161
C5—H5⋯O1^i^	0.95	2.34	3.263 (9)	165
C6—H6⋯O2^ii^	0.95	2.35	3.252 (11)	158
C9—H9⋯O4^iii^	0.95	2.28	3.188 (9)	161
C10—H10⋯O1	0.95	2.41	2.973 (8)	118
C21—H21⋯O1^iv^	0.95	2.44	3.175 (8)	134
C25—H25⋯O1^v^	0.95	2.44	3.285 (11)	149
C25—H25⋯O4^v^	0.95	2.39	3.255 (11)	151
C26—H26⋯O3^vi^	0.95	2.50	3.200 (8)	130
C28—H28⋯O4^vi^	0.95	2.46	3.367 (10)	159
C30—H30⋯O41^iii^	0.95	2.45	3.165 (12)	132

Symmetry codes: (i) 
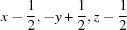
; (ii) 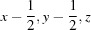; (iii) 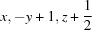; (iv) 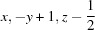; (v) 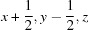; (vi) 
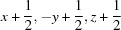
.

**Fig. 1.**
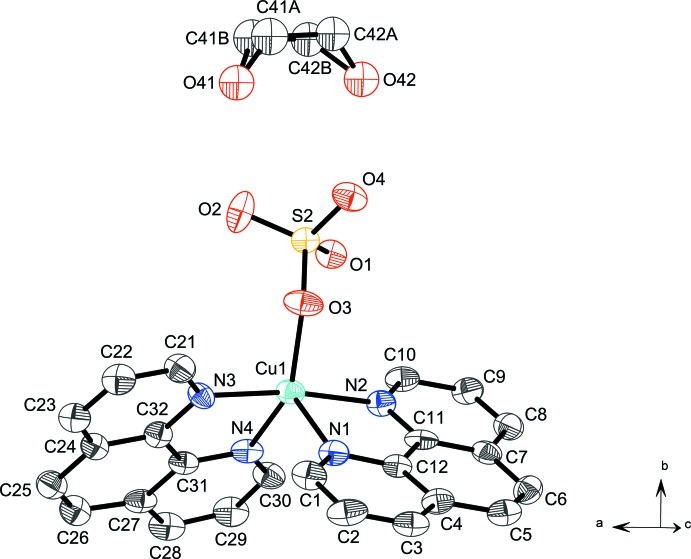
The asymmetric unit of I drawn with 50% probability for displacement ellipsoids. Hydrogen atoms are omitted for clarity.

**Fig. 2.**
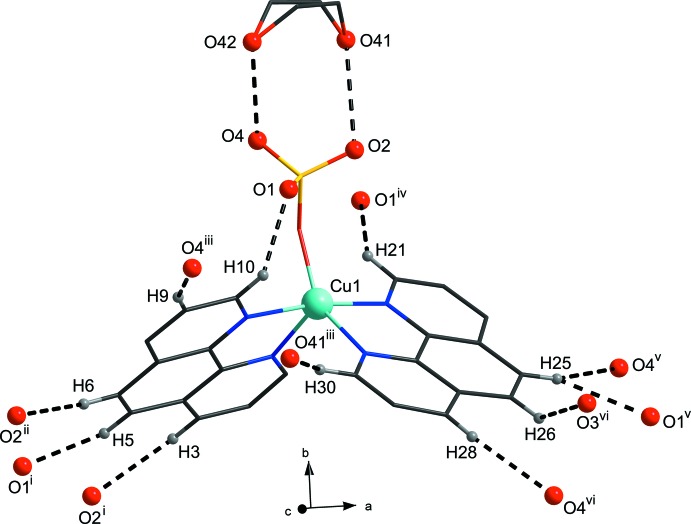
View of the hydrogen bonding interactions (dash lines). [Symmetry codes: (i) x - 1/2, -y + 1/2, z - 1/2; (ii) x - 1/2, y - 1/2, z; (iii) x, -y + 1, z + 1/2; (iv)x, -y + 1, z - 1/2; (v) x + 1/2, y - 1/2, z; (vi) x + 1/2, -y + 1/2, z + 1/2]

**Fig. 3.**
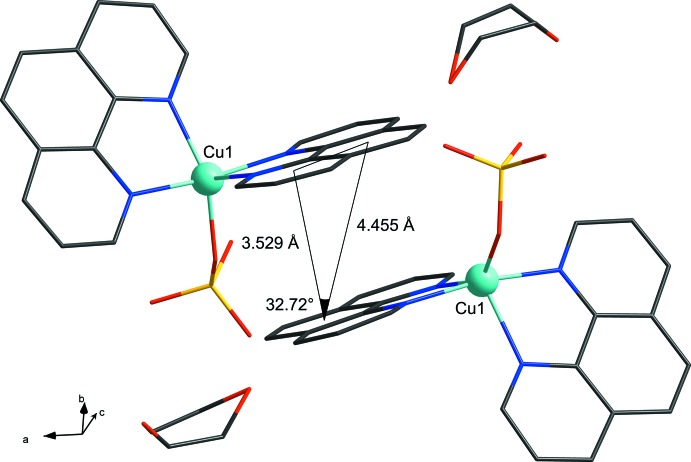
View of the π– π interactions between the adjacent o–phen molecules.

**Fig. 4.**
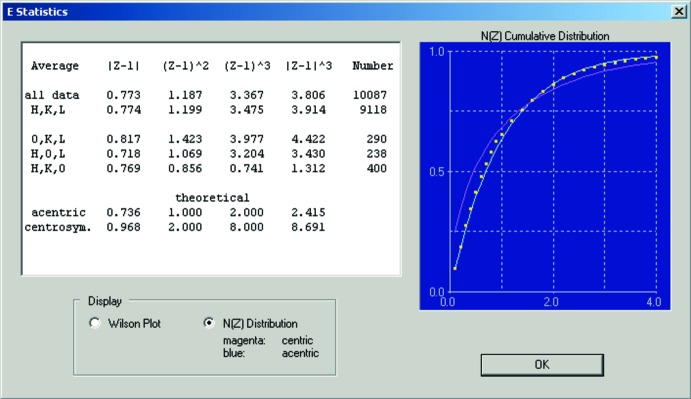
Normalized structure factor statistics. The inset graph shows the N(Z) distribution.

**Fig. 5.**
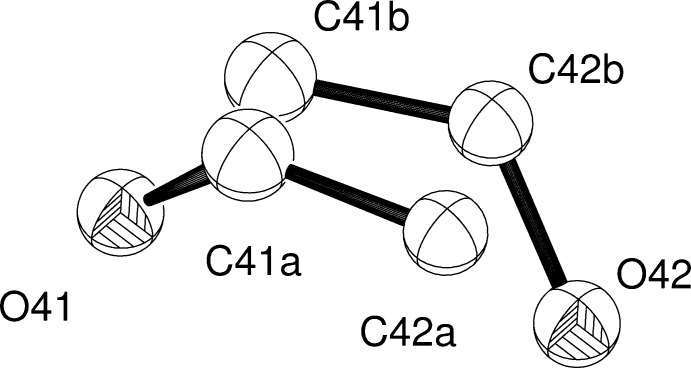
Arrangement of the two orientations of the disordered ethanol molecule. The major orientation (66%) is O41 C41a C42b. The minor orientation (34%) is O42 C42b C41b.

**Fig. 6.**
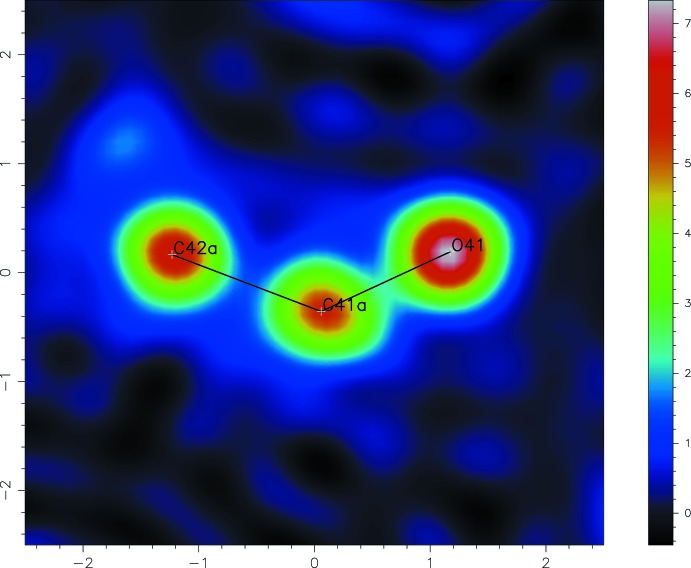
Fobs map calculated in the plane defined by O41 C41a C42a using all observed data. The x and y axes are labelled in Å and the scale is in eÅ-3.

**Fig. 7.**
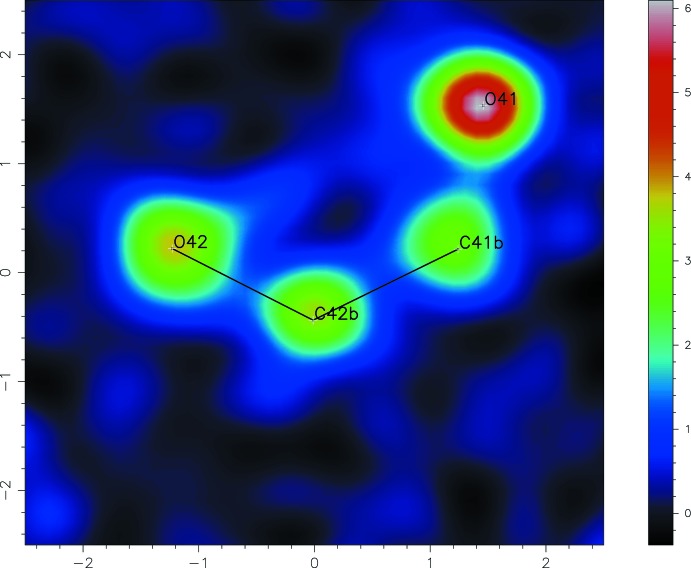
Fobs map calculated in the plane defined by O42 C42b C41b using all observed data. The x and y axes are labelled in Å and the scale is in eÅ-3.

## supplementary crystallographic information

### 1. Comment

The crystal structure of the title complex, [Cu(SO_4_)(C_12_H_8_N_2_)_2_]·C_2_H_6_O (I), is isostructural with the propane-1,2-diol and ethane-1,2-diol solvates, Cu(C_12_H_8_N_2_)_2_(SO_4_)·C_3_H_8_O_2_ (Zhong, 2011*a*) and [CuSO_4_(C_12_H_8_N_2_)_2_]·C_2_H_6_O_2_ (Zhong, 2011*b*). The neutral complex [Cu(SO_4_)(C_12_H_8_N_2_)_2_] in I is composed of a central Cu^II^ ion, coordinated by a single oxygen atom (O3) of the monodentate sulfato ligand, and two pairs of nitrogen atoms (N1, N2, N3 and N4) of two independent *N*,*N*'–chelating o–phen (see Fig. 1). Rather than the square pyramidal geometry described for some related complexes (Zhong, 2011*a*; Zhong, 2011*b*), the coordination about the Cu^II^ ion in I is better described as a trigonal bipyramid. In some previous reports, disorder of the sulfato ligand has introduced problems in the refinement. (*e.g* a Cu–O bond of length 1.4 Å (Zhong, 2011*a*)) We see no evidence for disorder in the sulfato ligand. The coordinating atoms N1, N4 and O3 are located in the trigonal bipyramidal plane with a summation of the three angles about the metal center close to 360°: O3—Cu1—N1 103.76 (18)°, O3—Cu1—N4 146.72 (19)° and N1—Cu1—N4 109.17 (14)°. The apical positions of the trigonal bipyramid are occupied by atoms N2 and N3 with the N2—Cu1—N3 angle of 170.74 (16)°. The Cu—O (1.947 (4) Å) and Cu—N (1.995 (5)–2.191 (6) Å) bond lengths in complex I are nontheless in good agreement with those reported for the relevant structures (Zhong, 2011*a*; Zhong, 2011*b*). The two independent chelating o–phen ligands anchored onto the same metal ion are oriented in a different planes with a slanting angle of 70.8 (1)° between the two molecular planes.

The spatial arrangement of each structural building motifs and the three–dimensional supramolecular assembly in I are regulated by the weak C—H···O hydrogen-bonding interactions (Fig. 2) in synergy with the π-π interactions (Fig. 3). Every oxygen atom, including those of the sulfato ligand, and the solvent molecule display C—H···O interactions with the chelating o–phen ligands, calculated by using the program *PLATON* (Spek, 2009). A close proximity between the sulfato oxygen atoms and the hydroxyl group of the solvent molecule suggests the possibility for the hydrogen bonding interactions between the two: O41···O2 2.8757 (89) Å and O42···O4 2.719 (16) Å. In addition to the hydrogen bonding interactions, the two adjacent *o*-phens exhibit the offset face-to-face π-π stacking with the inter–plane distance of 3.529 Å, centroid–to–centroid distance of 4.455 Å, and a displacement angle of 32.72°.

Systematic absences indicated a choice of space groups: *Cc* or *C*2/*c* and statistics of normalized structure factors suggested the structure was non-centric. The |*Z*-1| value of 0.773 for all data is close to the expected value for a non-centrosymmetric structure of 0.736. Similarly the N(*Z*) distribution provides a further indication suggests the structure is non-centric. Detailed E-statistics are contained within Figure 4.

Refinements in the two possible space group choices were perfomed and these clearly indicated that *C*2/*c* was incorrect. In particular, the wR2 was substantially better in the non-centric case. (wR2 = 0.1595 for all data in *Cc* and 0.2498 for all data in *C*2/*c*.) The C—C bond precision was better in the non-centric case (0.0115 compared with 0.0130 Å). This would not be the case if a strict centre of symmetry was present. *Cc* was therefore retained as the space group.

The comparison to other structure mentioned previously is important. Those similar structures reported in *Cc* (*eg* Zhong, 2011*b* and Zhong & Cai, 2013) have an ordered monodentate sulfate ligand. Those in *C*2/*c* (*eg* Wojciechowska *et al.*, 2011; Zhu *et al.*, 2006, and Zhong *et al.*, 2009) have an orderd bidentate sulfate ligand. Here the stable model in *Cc* has a mondentate sulfate with no ligand disorder, but the model in *C*2/*c* displays a disordered monodentate sulfate in contrast to the other reports. The refinement in *C*2/*c* is contained within the CIF for completeness but the crystal data and refinements indicate this is not the correct space group.

The crystal examined displayed racemic twinning. The refined twin fraction of the second component was 0.346 (19). This value is significantly different from 1/2, the value that would be expected if the compound was truly centrosymmetric and incorrectly refined in the space group *Cc*.

The ethanol solvent molecule is disordered over two positions, related by a rotation of approximately 180° about the C—C bond. The atoms of each orientation were identified in difference Fourier maps. The presence of ethanol is clear from these and the existence of two molecules in different orientations is apparent. Figure 5 shows the relationship between two orientations of the ethanol. Figures 6 and 7 show F_obs_ Fourier maps calculated using all observed data. From these the molecule can clearly be identified as ethanol, precluding any inclusion of dimethylsulfoxide or thiourea from the reaction mixture. The two orientations are present in the ratio 66.3:33.7 (10) %. For the major orientation, O41 forms a hydrogen bond to O2 while for the minor orientation, O42 forms a hydrogen bond to O4.

### 2. Experimental

The crystals of [Cu(SO_4_)(C_12_H_8_N_2_)_2_]·C_2_H_4_O (I) were unexpectedly obtained as a by-product during an attempt to synthesize copper complexes using mixing ligands of 1,10–phenanthroline (o–phen) and thiourea by a bilayer-diffusion method. In a typical experiment, two immiscible solutions A and B were first prepared. Solution A: CuSO_4_·5H_2_O (0.0499 g, 0.2 mmol; Fisher Scientific 99.55%) was dissolved in 4.0 ml dimethylsulfoxide (Riedel-de Haën 99.5%) in a small test tube (diameter of *ca* 13 mm). Solution B: 1,10-phenanthroline (o–phen; 0.0793 g, 0.4 mmol; QRëC 99.5%) and thiourea (0.0305 g, 0.4 mmol; Merck 99.0%) were dissolved in 4.0 ml e thanol (Merck 99.9%). Solution B was then gently added onto the surface of solution A. After 24 h, blue block shaped crystals were crystallized and isolated for single-crystal *X*-ray diffraction experiment.

### 3. Refinement

Hydrogen atoms were fitted using a riding model. The isotropic displacement factor for each hydrogen atom is 1.2 times that of the atom on which it rides.

### Crystal data

**Table:**

[Cu(SO_4_)(C_12_H_8_N_2_)_2_]·C_2_H_6_O	*F*(000) = 1156
*M**_r_* = 564.06	*D*_x_ = 1.591 Mg m^−^^3^
Monoclinic, *C**c*	Mo *K*α radiation, λ = 0.71073 Å
Hall symbol: C -2yc	Cell parameters from 11381 reflections
*a* = 17.5488 (14) Å	θ = 1.8–29.5°
*b* = 11.9360 (11) Å	µ = 1.06 mm^−^^1^
*c* = 13.0663 (9) Å	*T* = 150 K
β = 120.664 (5)°	Block, blue
*V* = 2354.2 (3) Å^3^	0.32 × 0.24 × 0.12 mm
*Z* = 4	

### Data collection

**Table:**

Stoe IPDS2 diffractometer	5771 independent reflections
Radiation source: fine-focus sealed tube	4259 reflections with *I* > 2σ(*I*)
Graphite monochromator	*R*_int_ = 0.070
Detector resolution: 6.67 pixels mm^-1^	θ_max_ = 29.2°, θ_min_ = 2.2°
ω scans	*h* = −24→23
Absorption correction: numerical (*X-AREA*; Stoe & Cie, 2002)	*k* = −15→16
*T*_min_ = 0.727, *T*_max_ = 0.883	*l* = −17→17
10087 measured reflections	

### Refinement

**Table:**

Refinement on *F*^2^	Secondary atom site location: difference Fourier map
Least-squares matrix: full	Hydrogen site location: inferred from neighbouring sites
*R*[*F*^2^ > 2σ(*F*^2^)] = 0.058	H-atom parameters constrained
*wR*(*F*^2^) = 0.160	*w* = 1/[σ^2^(*F*_o_^2^) + (0.0966*P*)^2^] where *P* = (*F*_o_^2^ + 2*F*_c_^2^)/3
*S* = 1.01	(Δ/σ)_max_ < 0.001
5771 reflections	Δρ_max_ = 1.02 e Å^−^^3^
331 parameters	Δρ_min_ = −1.09 e Å^−^^3^
8 restraints	Absolute structure: Flack (1983), 2594 Friedel pairs
Primary atom site location: structure-invariant direct methods	Absolute structure parameter: 0.346 (19)

### Special details

**Table:**

Geometry. All e.s.d.'s (except the e.s.d. in the dihedral angle between two l.s. planes) are estimated using the full covariance matrix. The cell e.s.d.'s are taken into account individually in the estimation of e.s.d.'s in distances, angles and torsion angles; correlations between e.s.d.'s in cell parameters are only used when they are defined by crystal symmetry. An approximate (isotropic) treatment of cell e.s.d.'s is used for estimating e.s.d.'s involving l.s. planes.
Refinement. Refinement of *F*^2^ against ALL reflections. The weighted *R*-factor *wR* and goodness of fit *S* are based on *F*^2^, conventional *R*-factors *R* are based on *F*, with *F* set to zero for negative *F*^2^. The threshold expression of *F*^2^ > σ(*F*^2^) is used only for calculating *R*-factors(gt) *etc*. and is not relevant to the choice of reflections for refinement. *R*-factors based on *F*^2^ are statistically about twice as large as those based on *F*, and *R*- factors based on ALL data will be even larger.

### Fractional atomic coordinates and isotropic or equivalent isotropic displacement parameters (Å^2^)

**Table:**

	*x*	*y*	*z*	*U*_iso_*/*U*_eq_	Occ. (<1)
N1	0.9825 (4)	0.1792 (4)	0.0032 (4)	0.0321 (12)	
C11	0.9177 (5)	0.2042 (4)	0.1237 (6)	0.0314 (14)	
C9	0.9264 (5)	0.2899 (5)	0.3232 (7)	0.0358 (16)	
H9	0.9317	0.3189	0.3942	0.043*	
C8	0.8537 (6)	0.2293 (5)	0.2455 (7)	0.0388 (16)	
H8	0.8073	0.2179	0.2616	0.047*	
C12	0.9124 (4)	0.1580 (5)	0.0166 (5)	0.0270 (12)	
C7	0.8467 (5)	0.1836 (5)	0.1419 (6)	0.0322 (13)	
C1	0.9812 (5)	0.1396 (6)	−0.0927 (6)	0.0398 (15)	
H1	1.0303	0.1539	−0.1028	0.048*	
C4	0.8389 (5)	0.0977 (5)	−0.0645 (6)	0.0341 (14)	
C6	0.7713 (5)	0.1197 (6)	0.0568 (6)	0.0363 (14)	
H6	0.7240	0.1053	0.0705	0.044*	
C5	0.7675 (5)	0.0797 (5)	−0.0437 (6)	0.0369 (15)	
H5	0.7166	0.0394	−0.1004	0.044*	
C2	0.9085 (6)	0.0763 (5)	−0.1810 (6)	0.0437 (19)	
H2	0.9091	0.0477	−0.2484	0.052*	
C10	0.9946 (5)	0.3089 (5)	0.2959 (6)	0.0346 (14)	
H10	1.0448	0.3526	0.3483	0.042*	
C3	0.8385 (5)	0.0578 (5)	−0.1665 (5)	0.0358 (14)	
H3	0.7886	0.0176	−0.2254	0.043*	
N4	1.1718 (4)	0.1823 (4)	0.2833 (4)	0.0291 (11)	
N3	1.1675 (4)	0.2753 (4)	0.0952 (5)	0.0289 (11)	
C28	1.3163 (5)	0.0529 (6)	0.4483 (6)	0.0386 (14)	
H28	1.3636	0.0072	0.5043	0.046*	
C27	1.3163 (5)	0.0950 (5)	0.3475 (6)	0.0324 (14)	
C22	1.2237 (6)	0.3009 (6)	−0.0345 (6)	0.0389 (16)	
H22	1.2157	0.3323	−0.1061	0.047*	
C21	1.1627 (5)	0.3212 (6)	−0.0013 (6)	0.0382 (15)	
H21	1.1147	0.3701	−0.0489	0.046*	
C30	1.1765 (5)	0.1415 (5)	0.3818 (6)	0.0351 (14)	
H30	1.1291	0.1567	0.3951	0.042*	
C24	1.3057 (4)	0.1865 (5)	0.1399 (6)	0.0313 (13)	
C29	1.2464 (5)	0.0790 (6)	0.4645 (6)	0.0386 (16)	
H29	1.2469	0.0533	0.5336	0.046*	
C23	1.2969 (6)	0.2347 (6)	0.0355 (6)	0.0367 (15)	
H23	1.3404	0.2219	0.0140	0.044*	
O3	1.0467 (3)	0.4360 (3)	0.0884 (3)	0.0413 (10)	
O4	0.9925 (3)	0.6192 (4)	0.0848 (4)	0.0398 (10)	
C32	1.2395 (5)	0.2082 (5)	0.1656 (6)	0.0298 (13)	
C31	1.2409 (4)	0.1608 (5)	0.2672 (5)	0.0278 (12)	
C26	1.3840 (5)	0.0775 (6)	0.3205 (6)	0.0377 (15)	
H26	1.4338	0.0337	0.3737	0.045*	
C25	1.3812 (5)	0.1204 (6)	0.2219 (6)	0.0383 (14)	
H25	1.4282	0.1069	0.2075	0.046*	
Cu1	1.07989 (4)	0.28399 (4)	0.14929 (5)	0.03031 (16)	
S2	1.06669 (9)	0.54426 (10)	0.15762 (10)	0.0283 (3)	
N2	0.9884 (4)	0.2662 (4)	0.1978 (5)	0.0306 (12)	
O1	1.0717 (3)	0.5217 (3)	0.2709 (3)	0.0329 (8)	
O2	1.1491 (3)	0.5917 (4)	0.1765 (4)	0.0494 (13)	
C41A	1.0959 (9)	0.8927 (10)	0.1088 (12)	0.059 (3)*	0.695 (15)
H41A	1.0757	0.9478	0.0434	0.071*	0.695 (15)
H41B	1.1497	0.9256	0.1768	0.071*	0.695 (15)
C42A	1.0326 (8)	0.8986 (9)	0.1425 (11)	0.050 (2)*	0.695 (15)
H42A	1.0491	0.9641	0.1962	0.060*	0.695 (15)
H42B	0.9763	0.9186	0.0696	0.060*	0.695 (15)
C41B	1.132 (2)	0.890 (2)	0.158 (3)	0.059 (3)*	0.305 (15)
H41C	1.1178	0.9632	0.1164	0.071*	0.305 (15)
H41D	1.1954	0.8923	0.2192	0.071*	0.305 (15)
C42B	1.0858 (15)	0.8890 (19)	0.221 (2)	0.050 (2)*	0.305 (15)
H42C	1.1305	0.8718	0.3044	0.060*	0.305 (15)
H42D	1.0663	0.9671	0.2197	0.060*	0.305 (15)
O41	1.1240 (5)	0.8127 (6)	0.0766 (7)	0.0523 (15)*	0.663 (10)
O42	1.0135 (10)	0.8216 (12)	0.1916 (14)	0.0523 (15)*	0.337 (10)

### Atomic displacement parameters (Å^2^)

**Table:**

	*U*^11^	*U*^22^	*U*^33^	*U*^12^	*U*^13^	*U*^23^
N1	0.044 (3)	0.028 (3)	0.030 (3)	0.001 (2)	0.023 (3)	0.001 (2)
C11	0.048 (4)	0.017 (3)	0.027 (3)	0.001 (3)	0.018 (3)	−0.002 (2)
C9	0.049 (4)	0.031 (3)	0.035 (4)	−0.004 (3)	0.027 (4)	−0.003 (2)
C8	0.052 (4)	0.032 (3)	0.048 (4)	0.006 (3)	0.037 (4)	0.007 (3)
C12	0.034 (3)	0.023 (3)	0.020 (2)	0.002 (2)	0.011 (3)	0.001 (2)
C7	0.042 (4)	0.024 (3)	0.032 (3)	0.008 (3)	0.021 (3)	0.008 (2)
C1	0.055 (4)	0.037 (4)	0.030 (3)	0.003 (3)	0.024 (3)	−0.001 (3)
C4	0.044 (4)	0.029 (3)	0.027 (3)	−0.001 (3)	0.017 (3)	0.003 (2)
C6	0.038 (4)	0.036 (3)	0.036 (3)	−0.002 (3)	0.020 (3)	0.004 (3)
C5	0.043 (4)	0.030 (3)	0.028 (3)	−0.003 (3)	0.011 (3)	0.000 (2)
C2	0.073 (6)	0.031 (3)	0.028 (3)	−0.008 (3)	0.027 (4)	−0.009 (3)
C10	0.048 (4)	0.027 (3)	0.027 (3)	−0.004 (3)	0.017 (3)	−0.007 (2)
C3	0.047 (4)	0.027 (3)	0.021 (3)	−0.001 (3)	0.008 (3)	−0.003 (2)
N4	0.039 (3)	0.024 (2)	0.017 (2)	0.001 (2)	0.009 (2)	0.0012 (18)
N3	0.031 (3)	0.029 (3)	0.027 (3)	0.009 (2)	0.015 (3)	0.0079 (19)
C28	0.045 (4)	0.034 (3)	0.024 (3)	0.002 (3)	0.007 (3)	0.000 (2)
C27	0.037 (4)	0.024 (3)	0.024 (3)	−0.004 (2)	0.006 (3)	−0.006 (2)
C22	0.053 (4)	0.042 (4)	0.027 (3)	0.001 (3)	0.024 (4)	0.006 (3)
C21	0.050 (4)	0.037 (3)	0.038 (4)	0.006 (3)	0.029 (4)	0.009 (3)
C30	0.048 (4)	0.031 (3)	0.031 (3)	0.008 (3)	0.023 (3)	0.009 (2)
C24	0.031 (3)	0.031 (3)	0.028 (3)	0.002 (2)	0.012 (3)	−0.001 (2)
C29	0.049 (4)	0.039 (4)	0.021 (3)	0.004 (3)	0.013 (3)	0.008 (3)
C23	0.046 (4)	0.037 (3)	0.030 (3)	0.001 (3)	0.021 (3)	0.000 (3)
O3	0.069 (3)	0.0277 (18)	0.030 (2)	0.0063 (19)	0.027 (2)	−0.0005 (15)
O4	0.041 (3)	0.036 (2)	0.027 (2)	0.0091 (19)	0.007 (2)	0.0044 (18)
C32	0.031 (3)	0.030 (3)	0.023 (3)	−0.003 (2)	0.010 (3)	−0.006 (2)
C31	0.033 (3)	0.023 (3)	0.020 (2)	0.000 (2)	0.008 (3)	−0.002 (2)
C26	0.034 (4)	0.040 (4)	0.028 (3)	0.004 (3)	0.008 (3)	0.000 (3)
C25	0.035 (4)	0.035 (3)	0.037 (3)	0.001 (3)	0.012 (3)	−0.004 (3)
Cu1	0.0389 (3)	0.0257 (2)	0.0295 (3)	0.0021 (4)	0.0197 (3)	0.0019 (3)
S2	0.0318 (8)	0.0280 (5)	0.0221 (6)	−0.0002 (6)	0.0116 (6)	0.0000 (5)
N2	0.040 (3)	0.029 (3)	0.027 (3)	−0.001 (2)	0.020 (3)	−0.0030 (19)
O1	0.038 (2)	0.0352 (18)	0.0220 (17)	0.0030 (17)	0.0128 (18)	0.0037 (15)
O2	0.034 (3)	0.075 (4)	0.040 (3)	−0.013 (2)	0.020 (2)	0.002 (2)

### Geometric parameters (Å, º)

**Table:**

N1—C1	1.329 (7)	C22—C21	1.366 (10)
N1—C12	1.352 (8)	C22—C23	1.383 (11)
N1—Cu1	2.191 (6)	C22—H22	0.9500
C11—N2	1.342 (9)	C21—H21	0.9500
C11—C7	1.405 (10)	C30—C29	1.370 (10)
C11—C12	1.462 (8)	C30—H30	0.9500
C9—C8	1.364 (11)	C24—C32	1.389 (9)
C9—C10	1.431 (10)	C24—C23	1.415 (9)
C9—H9	0.9500	C24—C25	1.441 (10)
C8—C7	1.406 (9)	C29—H29	0.9500
C8—H8	0.9500	C23—H23	0.9500
C12—C4	1.382 (10)	O3—S2	1.513 (4)
C7—C6	1.437 (10)	O3—Cu1	1.947 (4)
C1—C2	1.424 (11)	O4—S2	1.462 (5)
C1—H1	0.9500	C32—C31	1.431 (8)
C4—C3	1.412 (9)	C26—C25	1.363 (10)
C4—C5	1.429 (10)	C26—H26	0.9500
C6—C5	1.366 (9)	C25—H25	0.9500
C6—H6	0.9500	Cu1—N2	2.015 (5)
C5—H5	0.9500	S2—O2	1.453 (5)
C2—C3	1.354 (11)	S2—O1	1.463 (3)
C2—H2	0.9500	C41A—O41	1.242 (11)
C10—N2	1.331 (8)	C41A—C42A	1.391 (12)
C10—H10	0.9500	C41A—H41A	0.9900
C3—H3	0.9500	C41A—H41B	0.9900
N4—C30	1.340 (7)	C42A—O42	1.260 (13)
N4—C31	1.356 (8)	C42A—H42A	0.9900
N4—Cu1	2.064 (5)	C42A—H42B	0.9900
N3—C21	1.337 (8)	C41B—O41	1.360 (17)
N3—C32	1.378 (9)	C41B—C42B	1.411 (18)
N3—Cu1	1.995 (5)	C41B—H41C	0.9900
C28—C29	1.382 (10)	C41B—H41D	0.9900
C28—C27	1.410 (9)	C42B—O42	1.384 (17)
C28—H28	0.9500	C42B—H42C	0.9900
C27—C26	1.416 (10)	C42B—H42D	0.9900
C27—C31	1.433 (9)		
			
C1—N1—C12	118.4 (6)	C30—C29—C28	120.7 (6)
C1—N1—Cu1	131.0 (5)	C30—C29—H29	119.7
C12—N1—Cu1	110.5 (4)	C28—C29—H29	119.7
N2—C11—C7	123.2 (6)	C22—C23—C24	118.5 (6)
N2—C11—C12	118.9 (6)	C22—C23—H23	120.7
C7—C11—C12	117.9 (6)	C24—C23—H23	120.7
C8—C9—C10	118.9 (6)	S2—O3—Cu1	128.5 (2)
C8—C9—H9	120.6	N3—C32—C24	122.9 (6)
C10—C9—H9	120.6	N3—C32—C31	115.2 (5)
C9—C8—C7	120.8 (7)	C24—C32—C31	121.9 (6)
C9—C8—H8	119.6	N4—C31—C32	118.3 (6)
C7—C8—H8	119.6	N4—C31—C27	123.7 (5)
N1—C12—C4	123.6 (5)	C32—C31—C27	118.0 (6)
N1—C12—C11	115.8 (6)	C25—C26—C27	123.4 (7)
C4—C12—C11	120.6 (6)	C25—C26—H26	118.3
C11—C7—C8	116.5 (6)	C27—C26—H26	118.3
C11—C7—C6	120.5 (6)	C26—C25—C24	119.0 (7)
C8—C7—C6	123.0 (6)	C26—C25—H25	120.5
N1—C1—C2	122.0 (7)	C24—C25—H25	120.5
N1—C1—H1	119.0	O3—Cu1—N3	91.59 (18)
C2—C1—H1	119.0	O3—Cu1—N2	96.1 (2)
C12—C4—C3	116.9 (6)	N3—Cu1—N2	170.74 (16)
C12—C4—C5	119.9 (6)	O3—Cu1—N4	146.72 (19)
C3—C4—C5	123.1 (7)	N3—Cu1—N4	81.9 (2)
C5—C6—C7	120.3 (6)	N2—Cu1—N4	94.3 (2)
C5—C6—H6	119.9	O3—Cu1—N1	103.76 (18)
C7—C6—H6	119.9	N3—Cu1—N1	93.5 (2)
C6—C5—C4	120.8 (7)	N2—Cu1—N1	79.7 (2)
C6—C5—H5	119.6	N4—Cu1—N1	109.17 (14)
C4—C5—H5	119.6	O2—S2—O4	110.5 (3)
C3—C2—C1	118.4 (6)	O2—S2—O1	111.1 (3)
C3—C2—H2	120.8	O4—S2—O1	110.2 (2)
C1—C2—H2	120.8	O2—S2—O3	110.0 (3)
N2—C10—C9	120.8 (6)	O4—S2—O3	106.0 (3)
N2—C10—H10	119.6	O1—S2—O3	108.9 (2)
C9—C10—H10	119.6	C10—N2—C11	119.8 (6)
C2—C3—C4	120.6 (7)	C10—N2—Cu1	125.1 (5)
C2—C3—H3	119.7	C11—N2—Cu1	115.0 (4)
C4—C3—H3	119.7	O41—C41A—C42A	131.8 (11)
C30—N4—C31	117.3 (6)	O41—C41A—H41A	104.3
C30—N4—Cu1	131.8 (4)	C42A—C41A—H41A	104.3
C31—N4—Cu1	110.7 (4)	O41—C41A—H41B	104.3
C21—N3—C32	117.6 (5)	C42A—C41A—H41B	104.3
C21—N3—Cu1	128.5 (5)	H41A—C41A—H41B	105.6
C32—N3—Cu1	113.8 (4)	O42—C42A—C41A	126.0 (13)
C29—C28—C27	119.0 (6)	O42—C42A—H42A	105.8
C29—C28—H28	120.5	C41A—C42A—H42A	105.8
C27—C28—H28	120.5	O42—C42A—H42B	105.8
C28—C27—C26	125.5 (7)	C41A—C42A—H42B	105.8
C28—C27—C31	116.1 (6)	H42A—C42A—H42B	106.2
C26—C27—C31	118.4 (6)	O41—C41B—C42B	126 (2)
C21—C22—C23	120.4 (6)	O41—C41B—H41C	105.8
C21—C22—H22	119.8	C42B—C41B—H41C	105.8
C23—C22—H22	119.8	O41—C41B—H41D	105.8
N3—C21—C22	122.9 (7)	C42B—C41B—H41D	105.8
N3—C21—H21	118.5	H41C—C41B—H41D	106.2
C22—C21—H21	118.5	O42—C42B—C41B	124 (2)
N4—C30—C29	123.1 (6)	O42—C42B—H42C	106.3
N4—C30—H30	118.4	C41B—C42B—H42C	106.3
C29—C30—H30	118.4	O42—C42B—H42D	106.3
C32—C24—C23	117.6 (6)	C41B—C42B—H42D	106.3
C32—C24—C25	119.2 (6)	H42C—C42B—H42D	106.4
C23—C24—C25	123.1 (6)		

### Hydrogen-bond geometry (Å, º)

**Table:**

*D*—H···*A*	*D*—H	H···*A*	*D*···*A*	*D*—H···*A*
C3—H3···O2^i^	0.95	2.48	3.389 (9)	161
C5—H5···O1^i^	0.95	2.34	3.263 (9)	165
C6—H6···O2^ii^	0.95	2.35	3.252 (11)	158
C9—H9···O4^iii^	0.95	2.28	3.188 (9)	161
C10—H10···O1	0.95	2.41	2.973 (8)	118
C21—H21···O1^iv^	0.95	2.44	3.175 (8)	134
C25—H25···O1^v^	0.95	2.44	3.285 (11)	149
C25—H25···O4^v^	0.95	2.39	3.255 (11)	151
C26—H26···O3^vi^	0.95	2.50	3.200 (8)	130
C28—H28···O4^vi^	0.95	2.46	3.367 (10)	159
C30—H30···O41^iii^	0.95	2.45	3.165 (12)	132

Symmetry codes: (i) *x*−1/2, −*y*+1/2, *z*−1/2; (ii) *x*−1/2, *y*−1/2, *z*; (iii) *x*, −*y*+1, *z*+1/2; (iv) *x*, −*y*+1, *z*−1/2; (v) *x*+1/2, *y*−1/2, *z*; (vi) *x*+1/2, −*y*+1/2, *z*+1/2.
